# Differential Virus-Specific IFN-Gamma Producing T Cell Responses to Marek’s Disease Virus in Chickens With B19 and B21 MHC Haplotypes

**DOI:** 10.3389/fimmu.2021.784359

**Published:** 2022-01-13

**Authors:** Nitish Boodhoo, Shahriar Behboudi

**Affiliations:** ^1^ The Pirbright Institute, Woking, United Kingdom; ^2^ Faculty of Health and Medical Sciences, School of Veterinary Medicine, University of Surrey, Guilford, United Kingdom

**Keywords:** Marek’s disease virus, chicken, T cells, IFN-gamma, resistance, susceptible

## Abstract

Marek’s disease virus (MDV), the etiologic agent for Marek’s disease (MD), causes a deadly lymphoproliferative disease in chickens. Causes of the well-documented association between genetically defined lines of chicken and resistance to MD remain unknown. Here, the frequencies of IFN-gamma producing *pp38* and *MEQ*-specific T cell responses were determined in line N (B21 haplotype; MD-resistant) and line P2a (B19 haplotype, MD-susceptible) chickens after infection with vaccine and/or virulent (RB1B) strains of MDV using both standard *ex vivo* and cultured chIFN-gamma ELISPOT assays. Notably, MDV infection of naïve and vaccinated MD-resistant chickens induced higher frequencies of IFN-gamma producing MDV-specific T cell responses using the cultured and *ex vivo* ELISPOT assay, respectively. Remarkably, vaccination did not induce or boost *MEQ*-specific effector T cells in the susceptible chickens, while it boosted both *pp38*-and *MEQ*-specific response in resistant line. Taken together, our results revealed that there is a direct association between the magnitude of T cell responses to *pp38* and *MEQ* of MDV antigens and resistance to the disease.

## Introduction

Marek’s disease virus (MDV), Gallid herpesvirus 2 (GaHV-2), is the causative agent of Marek’s disease (MD), a deadly lymphoproliferative disease in chickens (1, 2). The clinical manifestation of MD is associated with immunosuppression (3), CD4^+^ lymphoma formation, infiltration of immune cells into various tissues, namely, central and peripheral nervous system (4), and metabolic dysregulation in the infected chickens (5–8). There is an association between the genetic background of chickens and the susceptibility/resistance to infectious diseases, namely, MD (9). Chicken line with B19 MHC haplotype is highly susceptible to MD, while B21 MHC haplotype chicken line is relatively resistant to MD. The resistant chicken line can become infected with the virulent MDV, but the infected birds do not develop the disease. Nevertheless, very virulent strains of MDV (e.g., RB1B) may cause MD in the MD-resistant line. MDV vaccine protects chickens from MD independent from their genetic background, however, the vaccine is more efficacious in the MD-resistant chickens (10). Notably, the vaccine-induced immunity fails to induce sterilizing immunity in chickens regardless of their genetic background and thus the infected chickens shed virus into the environment from skin and danders (1). This is thought to contribute to the emergence of more virulent MDV, which may break vaccine-induced immunity even in the genetically resistant chickens (1, 11).

Because of the cell-associated nature of MDV, T cell mediated immune response against MDV is believed to be crucial in the control of MD. Cytotoxic T cell responses to MDV antigens, namely, glycoproteins of MDV, the basic leucine zipper protein *MEQ* and MDV-encoded phosphoprotein *pp38* have been observed in both MD-resistant (B21 MHC haplotype; line N2a) and MD-susceptible (B19 MHC haplotype; line P2a). There were some differences in the magnitude and durability of T cell cytotoxicity to MDV antigens between the MD-resistant and MD susceptible chicken lines. Notably, CD8 T cell cytotoxicity was contracted faster but was not durable in the MD-susceptible chicken line (12). Moreover, a more potent cytotoxicity against *pp38* antigen was observed compared to the cells expressing *MEQ* antigen (13–15). Interferon-gamma (IFN-gamma) produced by antigen-specific T cells plays a key role in activation of anti-viral and anti-tumor immune responses, and it has been used as a possible surrogate marker of protection against infectious diseases and tumor development (16, 17).The administration of chicken IFN-gamma with MDV vaccine increases vaccine efficacy, suggesting that IFN-gamma may play an important role in providing protection against MD (18, 19).

We hypothesized that MDV infection, mounts more pronounced IFN-gamma producing MDV-specific T cell responses in the B21 haplotype line N chickens compared to that in the B19 MHC haplotype line P2a chickens. Here, the magnitude and kinetic of T cell responses to *pp38* and *MEQ* antigens were analyzed following experimental infection of naïve and vaccinated line N and line P2a chickens using both standard *ex vivo* and cultured chIFN-gamma ELISPOT assays. These two assays have been extensively used to detect two different antigen-specific human T cell populations (20, 21). Here, for the first time, these assays are simultaneously used to analyze antigen-specific T cell populations in chickens. Our results demonstrated that MDV infection induces a differential MDV-specific T cell response in these two chicken lines.

## Materials and Methods

### Ethics Statement

Animal experiments were approved by the ethical review committee at The Pirbright Institute (TPI) and the experiments were performed based on the guidelines and care approved by the UK government Home Office under project license PPL 30/3169. The personnel engaged in the procedures had acquired personal license from the UK Home Office.

### Synthetic Peptide Library

In total 66 peptides spanning the entire *MEQ* (GeneBank: ADN05237.1) protein and 55 peptides spanning the entire *pp38* (GenBank: ABR13155.1) protein respectively were synthesized by Mimotopes (United Kingdom). Peptides were 15 residues long and overlapped by 10 residues. Peptide were dissolved in DMSO, and peptide pools (1 mM) were prepared at 10 peptides per pool for a total of 7 and 6 peptide pools for *MEQ* and *pp38* respectively.

### Virus Preparation

Virus stocks were prepared as a 3rd passage virus for both the pathogenic (GaHV-2: RB1B) and vaccine strain (CVI988/Rispens) as a cell-associated stock in chicken embryonic fibroblast cells (CEFs) generated from ten-day-old mixed-sex specific pathogen free (SPF) Valo eggs (Valo Biomedia GmbH). The virus stocks were titrated on fresh CEFs based on anti-gB mAb (HB-3) staining and stored in liquid nitrogen. Commercial CVI988/Rispens vaccine virus (Nobilis Rismavac) was obtained from Intervet.

### Animal Experiments

**Figure 1:**
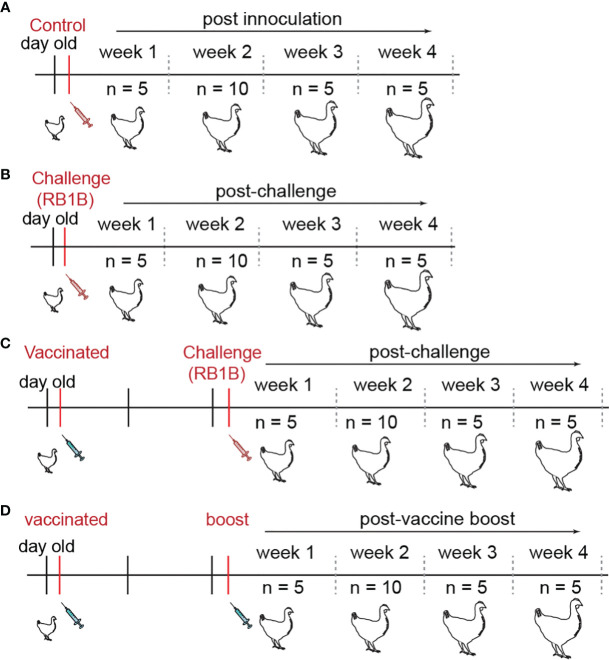
Chicken infection models developed to study T cell mediated immune responses. Schematic representation of experimental design to study cell mediated immune response to MDV in both line N (n = 100) and line P2a (n = 100) chickens. Day old chickens were administered with **(A)** non-infected chicken embryonated fibroblasts (control group; n = 25), **(B)** virulent MDV (RB1B; 1,000 PFU/chicken) (challenge group; n = 25), or vaccine strain of MDV (CVI988/RISPENS; 1,000 PFU/chicken). The latter group were either **(C)** infected with the virulent MDV (vaccine-challenge group; n = 25) or **(D)** boosted with the MDV vaccine two weeks later (vaccine-boost group; n = 25). The birds were sacrificed on weeks 1, 2, 3, and 4 post the last inoculation and the frequencies of IFN-gamma producing virus-specific T cells were determined.

In total, one hundred genetically defined mixed sex SPF chicken line N (MD-resistant; B21 MHC haplotype) and one hundred chicken line P2a (MD-susceptible; B19 MHC haplotype) were reared at The Pirbright Institute or purchased from the National Avian Research Facility (NARF) at University of Edinburgh. Day old chicks were group-housed throughout the experiment in specific pathogen free filtered-air positive pressure rooms on floor pens with wood shaving. Group-housed chickens had *ad libitum* access to water and commercial feed. Line N (MD-resistant; B21 haplotype) and Line P2a (MD-susceptible; B19 haplotype) chickens were either mock inoculated (non-infected CEF), challenged (RB1B; 1,000 pfu/chicken) or vaccinated (CVI988/RISPENS; 1,000 pfu/chicken) at 1 day of age *via* intra-abdominal route and the vaccinated group were either boosted (CVI988/RISPENS; 1,000 pfu/chicken) or challenged (RB1B; 1,000 pfu/chicken) 2 weeks later *via* intra-abdominal route. Splenocytes were harvested at 1, 2, 3, and 4 weeks post-vaccination/infection for further analysis (**Figure 1**).

### Spleen Mononuclear Cell Preparation

Splenocytes were isolated from spleens of chickens as previously described (4). In brief, spleens were crushed onto 40-μm BD cell strainers (BD Biosciences, UK), and the collected cells were layered on LymphoprepTM (Axis-shield PoC AS, Norway) density-gradient centrifugation, and centrifuged at 2,100 rpm for 20 min to allow the separation of mononuclear cells. Mononuclear cells were removed from the interface, and washed in cell culture media at 1,500 rpm for 5 min. Finally, the splenocytes were re-suspended in RPMI 1640 medium containing 10% fetal bovine serum (Sigma-Aldrich, Dorset, UK), penicillin (10 U/ml), and streptomycin (10 μg/ml) at a density of 5 × 10^6^ cells/ml.

### *Ex Vivo* Chicken IFN-Gamma ELISPOT Assay

*MEQ* or *pp38*-specific effector T cell response was evaluated based on the frequencies of IFN-gamma producing T cells using a chicken IFN-gamma ELISPOT assay. In brief, MAIPS4510 MultiScreenTM-IP 96 well plates (Millipore, UK) were incubated overnight at 4°C with 2 μg/ml mouse anti-chIFN-gamma capture antibody (ThermoFisher Scientific, UK) for 18 h. Mononuclear cells were seeded in triplicates at a rate of 3.3 × 10^5^ cells per well and the cells were stimulated with all *pp38*, *MEQ* derived peptide pools [containing 1 μM of each individual peptide; 10 peptides in each peptide pool) or an irrelevant peptide (Influenza H5_246–260_)]. In each assay, some cells were also incubated with diluent (negative control), or Phorbol Myristate Acetate (PMA; 50 ng/ml) plus Ionomycin (ION; 1 μg/ml) (positive control); (Millipore-Sigma, Dorset, UK), at 41°C and 5% CO_2_ overnight. Next day plates were washed twice with SQ water and three times with washing buffer (PBS + 0.1% Tween 20). Plates were subsequently incubated with detection antibody (1 μg/ml of anti-chicken IFN-gamma biotinylated antibody), followed by Streptavidin-HRP (1/1,250). The assay was developed in the presence of 3-Amino-9-ethylcarbazole (AEC) substrate solution (BD Biosciences, UK). The frequency of IFN-gamma producing T cells (spots forming units; SFU) was counted using an automated ELISPOT reader. ELISPOT wells in positive controls (PMA + Ionomycin) were black out and uncountable. The SFU was calculated by subtracting the number of spots obtained in the non-stimulated control wells or irrelevant peptide (H5_246–260_).

### Cultured IFN-Gamma ELISPOT Assay

The cultured ELISPOT assay has been extensively used to detect antigen-specific IFN-gamma-producing human T cells by us (22–27) and others (20, 21, 28), and here we adopted this method to analyze MDV-specific-IFN-gamma producing responses in chickens. The cultured method is used to demonstrate *in vitro* induction and expansion of effector T cells, akin to identification of antigen-specific memory T cell where the use of specific phenotypic markers is lacking. Mononuclear cells isolated from spleens of chickens from different experimental groups at 1-, 2-, 3-, and 4-weeks post challenge or vaccine boost were seeded (1 × 10^6^ cells/200 ul) in 96-well plate (round bottom) and were incubated at 41°C) in cell-culture media supplemented with 10% FCS stimulated with recombinant chicken IL-2 (rIL-2; 2 units per 100 μl) in combination with medium alone, vehicle (DMSO) or *pp38*-derived peptide pools 1 and 4 for line P2a and pool 4 for line N; each individual peptide was at a final concentration of 2 μmol/ml. One hundred microliters of cell culture media were refreshed every two days with rIL-2 with/without specific peptide pools until day 9 *in vitro*. At day 9, the expanded T cell lines were harvested, washed twice in cell culture media and viability (50% total live cells) were determined. Cell cultures were adjusted based on viability and seeded (3.3 × 10^5^ cells/per well) in MAIPS4510 MultiScreenTM-IP 96 well plates to determine the frequencies of antigen specific IFN-gamma producing T cells using chicken IFN-gamma ELISPOT assay.

### qPCR to Amplify MDV Genes

Feather pulp DNA samples from week 2 old Line N and Line P2a were isolated using the DNeasy-96 kit (Qiagen, Ca.) according to the manufacturer’s instructions. DNA samples were measured using a nanodrop and all samples were adjusted to 50 ng/ul prior to use. A master-mix was prepared: primers Meq-FP (GGTCTGGTGGTTTCCAGGTGA; 0.4 μM) and Meq-RP (GCATAGACGATGTGCTGCTGA; 0.4 μM), Meq probes (AGACCCTGATGATCCGCATTGCGACT; 0.2 μM, 5′FAM-3′BHQ1, Eurogentec), ovotransferrin (ovo) forward (CACTGCCACTGGGCTCTGT; 0.4 μM) and reverse primers (GCAATGGCAATAAACCTCCAA; 0.4 μM), and ovo probe (AGTCTGGAGAAGTCTGTGCAGCCTCCA; 0.2 μM, 5′Yakima Yellow-3′TAMRA, Eurogentec) and ABsolute Blue^®^ q-PCR Low Rox master-mi (Thermo Fisher Scientific, Ca.). A standard curve generated for both Meq (10-fold serial dilutions prepared from plasmid construct with Meq target) and the ovo gene (10-fold serial dilutions prepared from plasmid construct with ovo target) were used to normalize DNA samples and to quantify MDV genomes. All reactions were performed in triplicates to detect both Meq and the chicken ovotransferrin (ovo) gene on an ABI7500 system (Applied Biosystems) using standard conditions. MDV genomes were normalized and are reported as mean viral genome copy number/mg of tissue.

### Statistical Analysis

ELISPOT SFU data were adjusted to 10^6^ cells. Quantification was performed using Graph Pad Prism 6 for windows. All data were analyzed by one-way ANOVA or Wilcoxon and Mann–Whitney non-parametric to test significance and presented as mean + SD. Results were considered statistically significant at *P <*0.05 (*).

An immunological response/responder was defined as a 2-fold increase in the frequency of cytokine-producing cells above control peptide/pools.

## Results

### Infection With MDV Induces Virus-Specific Effector T Cells in Both MD-Resistant and MD-Susceptible Naïve Chickens

Splenocytes were isolated from the experimental groups (**Figure 1**) at 1-, 2-, 3-, and 4-weeks post the second inoculation (at least 5 birds per group for weeks 1, 3, and 4; at least 10 birds per group for week 2), and the frequencies of antigen-specific-IFN−gamma-producing effector T cells in response to the overlapping *MEQ* and *pp38* derived peptides were assessed using the standard *ex vivo* and cultured chIFN-gamma ELISPOT assays. As expected, no typical MD clinical (e.g., paralysis) or gross pathological signs (e.g., lymphoma formation) were observed up to 4 wpi in the experimental groups. Based on our previous observations, the severe clinical and gross pathological signs become detectable at 8 wpi. However, detection of MDV *MEQ* gene, using qPCR, in feather pulps of MDV-infected chickens at 2 wpi confirmed successful MDV infection in the experimental groups. In detail, MDV copy numbers were 22,400 ± 6,707 in the vaccine-challenge group and 32,112 ± 7,918 in the challenge group per 10^4^ cells.

**Figure 2:**
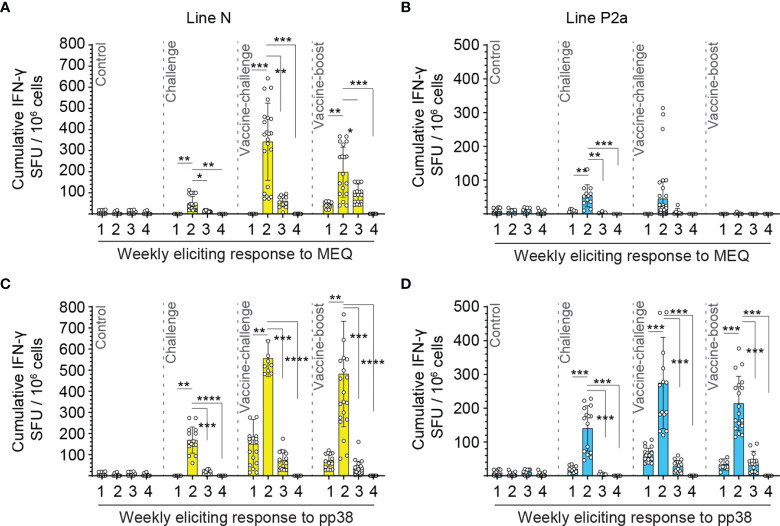
*MEQ* and *pp38*-specific T cells are peaked at two weeks post inoculation in both line N and line P2a chickens. Mononuclear cells were isolated from spleens of the experimental groups at weeks 1, 2, 3, and 4 post last inoculation and the frequencies of antigen-specific IFN-gamma producing T cells in response to *MEQ* or *pp38* derived peptide pools were determine in line N (yellow bars) **(A, C)** and line P2a (blue bars) **(B, D)** chickens using an *ex vivo* chicken IFN-gamma ELISPOT assay. The frequencies of IFN-gamma producing cells are presented as SFU in 10^6^ splenocytes (in triplicate for each bird) and shown as mean ± SD of each ELISPOT well. Splenocytes. Non-parametric Wilcoxon tests (Mann–Whitney) was used to assess normal distribution and test significance. * (*P* ≤ 0.05), ** (*P* ≤ 0.01), *** (*P* ≤ 0.005) and **** (*P* ≤ 0.001) indicates as statistically significant as corrected from the mock-infected control chickens.

Using *ex vivo* ELISPOT assay, no MDV-specific-IFN-gamma-producing T cell response was detected in the control group (**Figure 2**). In contrast, MDV infection induced *MEQ* and *pp38*-specific-IFN-gamma-producing T cell responses in both line N (**Figures 2A, C** **)** and line P2a (**Figures 2B, D** **)** chickens. The peaks of virus-specific-IFN-gamma-producing effector T cell responses were observed at 2 wpi in all the experimental groups in both line N and P2a chickens except for the vaccine-boost group from line P2a chickens which did not show any detectable response. The frequencies of virus-specific-IFN-gamma-producing effector T cells were reduced at 3 wpi and became undetectable at 4 wpi (**Figure 2**).

### Infection of the Vaccinated Line N Chickens Elicits a Greater Magnitude of *pp38* and *MEQ*-Specific T Cell Responses Compared to the Vaccinated Line P2a Chickens

**Figure 3:**
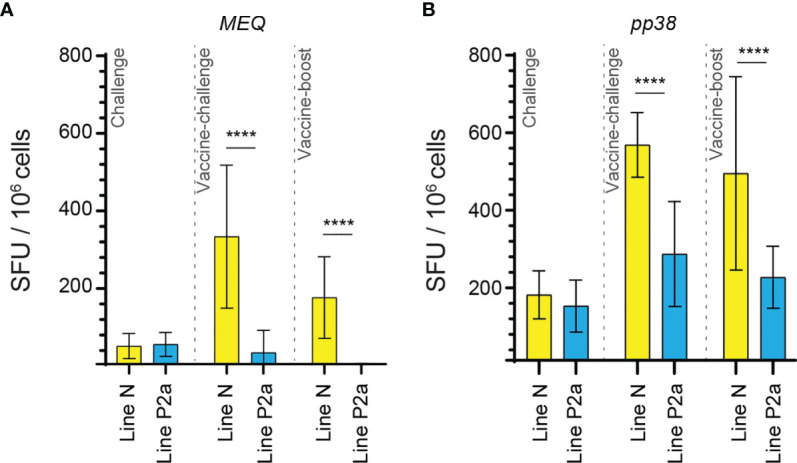
Higher frequencies of *MEQ* and *pp38*-specific T cell responses in line N chickens compared to line P2a chickens from vaccine-challenge and vaccine-boost groups at week 2 post inoculation. The frequencies of **(A)** *MEQ* **(B)** *pp38*-specific IFN-gamma producing T cell responses in line N (yellow bars) and line P2a (blue bars) chickens within the challenge, vaccine-challenge, and vaccine-boost groups are shown at 2 weeks post last inoculation using standard *ex vivo* ELISPOT assay. The results are presented as SFU per 10^6^ cells and shown as mean ± SD. Non-parametric Wilcoxon tests (Mann–Whitney) was used to assess normal distribution and test significance. **** (*P* ≤0.001) indicates as statistically significant.

**Figure 4:**
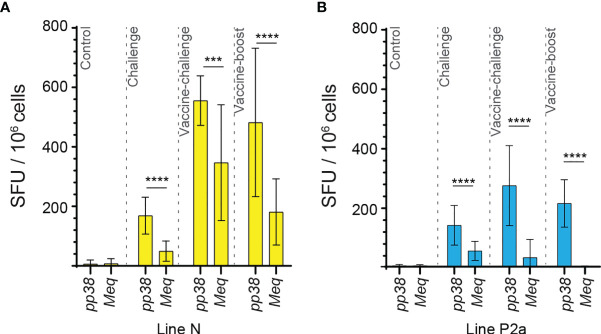
High frequencies of IFN-gamma producing T cells are detected against *pp38* compared to *MEQ* in the experimental groups of both chicken lines at 2 weeks post the last inoculation. The frequencies of IFN-gamma producing T cells recognizing *MEQ* and *pp38*-derived peptide pools, detected using an *ex vivo* chIFN-gamma ELSIPOT assay, are shown in **(A)** line N (yellow) and **(B)** line P2a (blue) chickens at 2 weeks post inoculation within the experimental groups. The results are presented as SFU per 10^6^ cells and shown as mean ± SD. Non-parametric Wilcoxon tests (Mann–Whitney) was used to assess normal distribution and test significance. *** (*P* ≤ 0.005) and **** (*P* ≤ 0.001) indicates as statistically significant.

**Figure 5:**
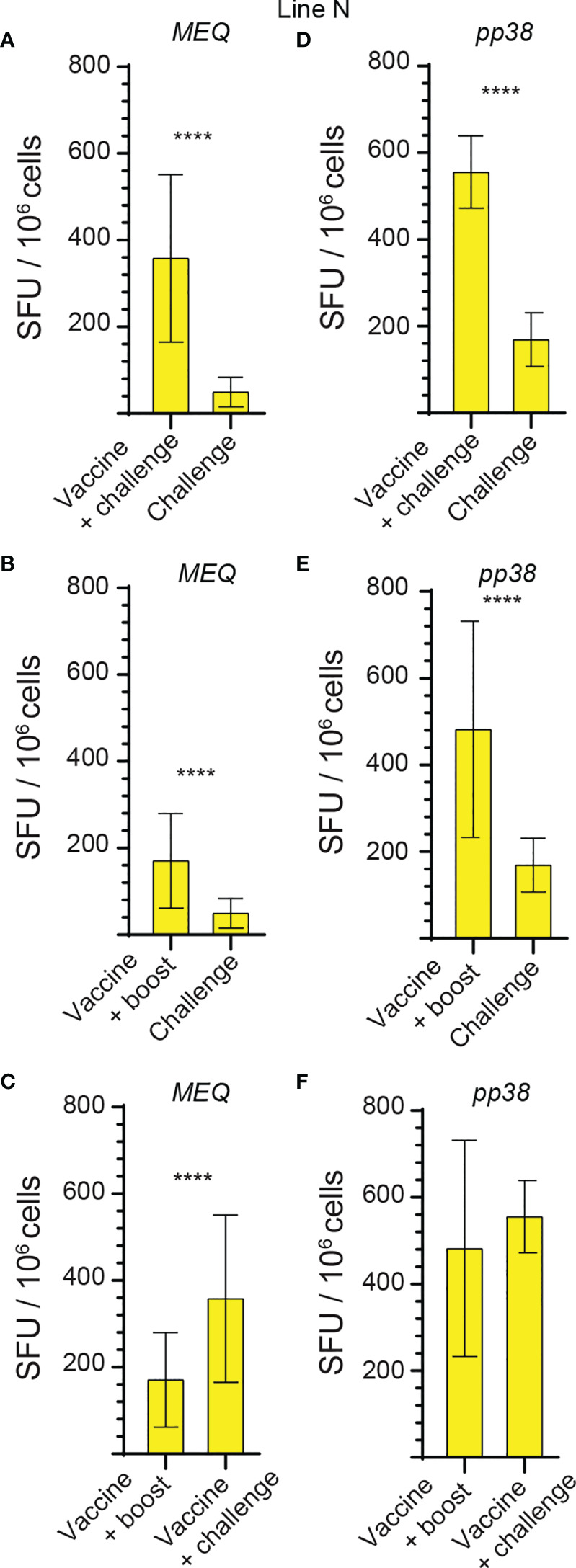
Higher frequencies of *MEQ* and *pp38*-specific T cell responses in vaccine-challenge and vaccine-boost groups in line N chickens at 2 weeks post last inoculation. The frequencies of antigen-specific IFN-gamma producing T cells, detected using a standard *ex vivo* ELISPOT assay, to **(A–C)** *MEQ* and **(D–F)** *pp38* derived peptide pools in the different experimental groups of line N chickens at 2 weeks post last inoculation are graphed. The results are presented as SFU per 10^6^ cells and shown as mean ± SD. Non-parametric Wilcoxon tests (Mann–Whitney) was used to assess normal distribution and test significance. **** (*P* ≤ 0.001) indicates as statistically significant.

**Figure 6:**
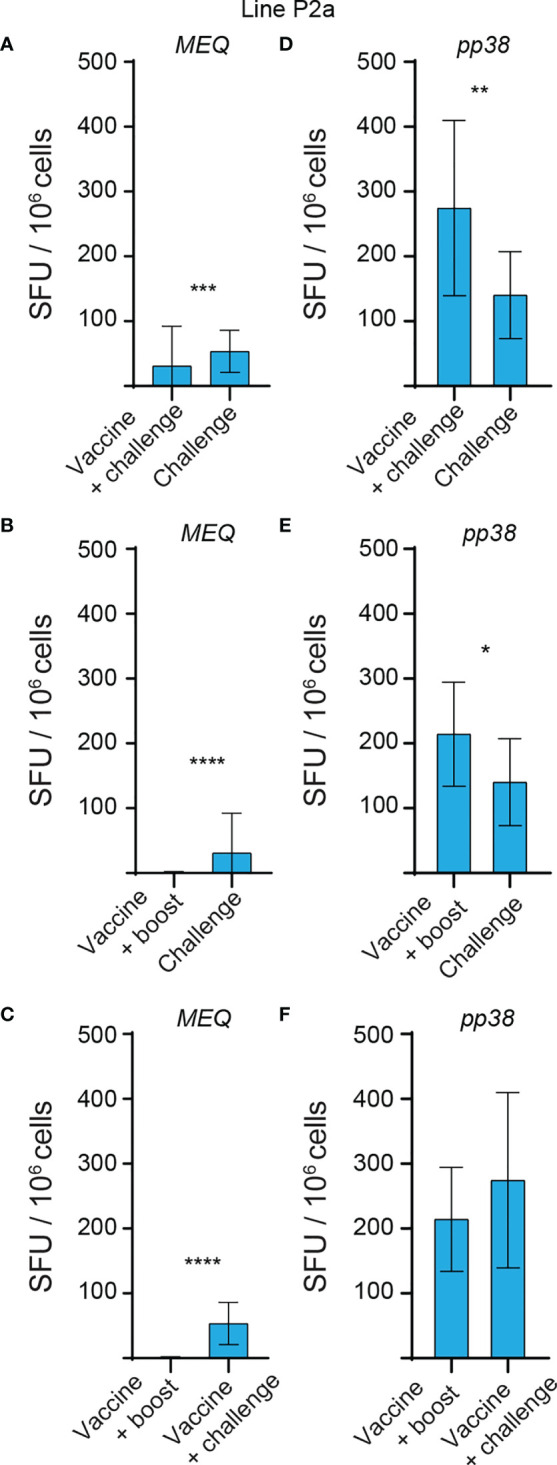
No *MEQ*-specific IFN-gamma producing T cell response is induced in vaccine-boost group in line P2a chickens at 2 weeks post last inoculation. The frequencies of antigen-specific IFN-gamma producing T cells, detected using a standard *ex vivo* ELISPOT assay, to **(A–C)** *MEQ* and **(D–F)** *pp38* derived peptide pools in the different experimental groups of line P2a chickens at 2 weeks post last inoculation are shown. The results are presented as SFU per 10^6^ cells and shown as mean ± SD. Non-parametric Wilcoxon tests (Mann–Whitney) was used to assess normal distribution and test significance. * (*P* ≤ 0.05), ** (*P* ≤ 0.01), *** (*P* ≤ 0.005) and **** (*P* ≤ 0.001) indicates as statistically significant.

The generated data above, obtained using *ex vivo* chIFN-gamma ELISPOT assay, were replotted for easier visual comparison of the *MEQ* and *pp38-*specific T cell responses at 2 wpi (peak of response; at least 10 birds per group) in line N and line P2a chickens in the different experimental groups. In the challenge group, there was no difference in magnitudes of the *MEQ* (**Figures 3A**) and *pp38-*specific T cell responses (**Figure 3B**) between line N and line P2a chickens. In contrast, in the vaccine-challenge and vaccine-boost groups, the frequencies of T cells against *MEQ* (p <0.001) (**Figure 3A**) and *pp38* (p <0.001) (**Figure 3B**) were higher in the line N chickens compared to that in line P2a chickens. Interestingly, MDV vaccine induced and boosted *MEQ*-specific T cell response in line N, but not in in line P2a, chickens (**Figure 3**). Greater frequencies of *pp38*-specific T cell responses compared to *MEQ*-specific T cell responses were detected (p <0.05) in challenge, vaccine-challenge, and vaccine-boost groups in both chicken lines, (**Figures 4A, B** **)**. In line N chickens, (a) higher frequencies of the *pp38-* and *MEQ*-specific T cells were detected in the vaccine-challenge or vaccine-boost groups compared to the challenged group; (b) higher frequencies of *MEQ*-specific-IFN-gamma-producing T cells were detected in the vaccine-challenge group compared to vaccine-boost group; and (c) similar levels of the *pp38*-specific T cells in the vaccine-challenge and vaccine-boost groups were observed (**Figures 5A–F**). There is no difference in the magnitude of *MEQ*-specific T cell response between the challenge and vaccine-challenge groups (**Figure 6A**). In the line P2a chickens, (a) no *MEQ*-specific-IFN-gamma-producing effector T cells were detected in the vaccine-boost group (**Figures 6B, C** **)**, (b) higher frequencies of *pp38*-specific-IFN-gamma-producing effector T cells were detected in the vaccine-challenge and vaccine-boost groups compared to the challenge group (**Figures 6D, E** **)**; (c) no difference in the frequencies of *pp38*-specific-IFN-gamma-producing T cells was observed between the vaccine-challenge and vaccine-boost groups (**Figure 6F**); Critically, in the vaccine-boost and vaccine-challenge groups, significantly higher frequencies of *pp38* and *MEQ*-specific-IFN-gamma-producing T cells were detected in the line N chickens compared to the line P2a chickens (**Figures 3A, B** **)**.

### Expanded Breadth of MDV-Specific T Cells in the Line N Chickens

**Figure 7:**
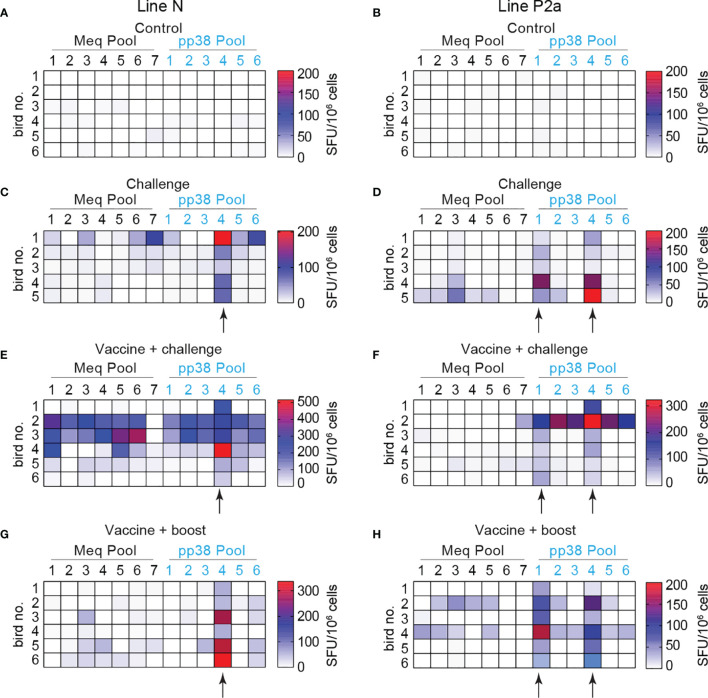
Expanded breadth of effector T cells in vaccine-challenge group of line N chickens. Splenocytes were isolated 2 weeks after the last inoculation from the experimental groups; control **(A, B)**, challenge **(C, D)**, vaccine-challenge **(E, F)** and vaccine-boost **(G, H)** from both line N and P2a chickens. The frequencies of IFN-gamma producing T cells recognizing 7 different peptide pools spanning entire *MEQ* and 6 different peptide pools spanning entire *pp38* were determined using an *ex vivo* chIFN-gamma ELSIPOT assay and the results are presented as a heat map. Black arrows indicate the peptide pools with potential immunodominant epitopes. All ELISPOT assays were performed in triplicates and results are representative of 5–6 chickens within each experimental group from each chicken line. Arrows shows the peptide pools from *pp38* derived peptides activating IFN-gamma production from splenocytes of most of the line N and line P2a chickens within the challenge, vaccine-challenge, and vaccine-boost groups.

The frequencies of IFN-gamma-producing effector T cells to seven *MEQ*-derived peptide pools and six *pp38*-derived peptide pools were analyzed using an *ex vivo* chIFN-gamma ELISPOT assay (**Table 1**). Splenocytes were isolated from the experimental groups (control, challenge, vaccine-challenge, and vaccine-boost) at week 2 post the last inoculation and the breadth of *pp38* and *MEQ-*specific-IFN-gamma-producing T response from 46 chickens was determined (**Figures 7A–H**). The positive response was considered as twice as background. In the vaccine-challenge groups, forty-one measurements were positive in line N chickens, while only 20 measurements were above the background in line P2a chickens. In contrast, a narrow *pp38* and *MEQ*-specific-IFN-gamma-producing T cell response was induced in the infected line N (**Figure 7C**) and line P2a chickens (**Figure 7D**) (20 measurements being positive in each chicken line). Taken together, the results suggest that the breadth of T cell response is associated with specific genetic background in the vaccine-challenge group.

**Table 1:** 15 mer. with 5 overlapping MDV *MEQ* and *pp38* peptide pools.

Peptide pool		Peptide sequences					
*MEQ*	1	1	MSQEPEPGAMPYSPA	5	LDLSLGSTSRRKKRK		
		2	EPGAMPYSPADDPSP	6	GSTSRRKKRKSHDIP	9	NSPSKHPFPDGLSEE
		3	PYSPADDPSPLDLSL	7	RKKRKSHDIPNSPSK	10	HPFPDGLSEEEKQKL
		4	DDPSPLDLSLGSTSR	8	SHDIPNSPSKHPFPD		
	2	1	GLSEEEKQKLERRRK	5	ARRRRRKQTDYVDKL		
		2	EKQKLERRRKRNRDA	6	RKQTDYVDKLHEACE	9	ELQRANEHLRKEIRD
		3	ERRRKRNRDAARRRR	7	YVDKLHEACEELQRA	10	NEHLRKEIRDLRTEC
		4	RNRDAARRRRRKQTD	8	HEACEELQRANEHLR		
	3	1	KEIRDLRTECTSLRV	5	EPVCPMAVPLTVTLG		
		2	LRTECTSLRVQLACH	6	MAVPLTVTLGLLTTP	9	HDPVPEPPICTPPPP
		3	TSLRVQLACHEPVCP	7	TVTLGLLTTPHDPVP	10	EPPICTPPPPSPDEP
		4	QLACHEPVCPMAVPL	8	LLTTPHDPVPEPPIC		
	4	1	TPPPPSPDEPNAPHC	5	PICTPPPPDTEELCA		
		2	SPDEPNAPHCSGSQP	6	PPPDTEELCAQLCST	9	PPPPISTPHIIYAPG
		3	NAPHCSGSQPPICTP	7	EELCAQLCSTPPPPI	10	STPHIIYAPGPSPLQ
		4	SGSQPPICTPPPPDT	8	QLCSTPPPPISTPHI		
	5	1	IYAPGPSPLQPPICT	5	AEELCAQLCSTPPPP		
		2	PSPLQPPICTPAPPD	6	AQLCSTPPPPICTPH	9	SLFCPPQPPSPEGIF
		3	PPICTPAPPDAEELC	7	TPPPPICTPHSLFCP	10	PQPPSPEGIFPALCP
		4	PAPPDAEELCAQLCS	8	ICTPHSLFCPPQPPS		
	6	1	PEGIFPALCPVTEPC	5	GTVYAQLCPVGQVPL		
		2	PALCPVTEPCTPPSP	6	QLCPVGQVPLFTPSP	9	PHPAPEPERLYARLT
		3	VTEPCTPPSPGTVYA	7	GQVPLFTPSPPHPAP	10	EPERLYARLTEDPEQ
		4	TPPSPGTVYAQLCPV	8	FTPSPPHPAPEPERL		
	7	1	YARLTEDPEQDSLYS	3	DSLYSGQIYTQFPSD	5	QFPSDTQSTVWWFPG
		2	EDPEQDSLYSGQIYT	4	GQIYTQFPSDTQSTV	6	DTQSTVWWFPGDGRP
*pp38*	1	1	MEFEAEHEGLTASWV	5	GGKGAEGRAGVADEA		
		2	EHEGLTASWVAPAPQ	6	EGRAGVADEAGHGKT	9	EAECAEDGEKCGDAE
		3	TASWVAPAPQGGKGA	7	VADEAGHGKTEAECA	10	EDGEKCGDAEMSALD
		4	APAPQGGKGAEGRAG	8	GHGKTEAECAEDGEK		
	2	1	CGDAEMSALDRVQRD	5	SPPPHSGVTGKGAIP		
		2	MSALDRVQRDRWRFS	6	SGVTGKGAIPIKGDG	9	KAIECQELTGEGEWL
		3	RVQRDRWRFSSPPPH	7	KGAIPIKGDGKAIEC	10	QELTGEGEWLSQWEE
		4	RWRFSSPPPHSGVTG	8	IKGDGKAIECQELTG		
	3	1	EGEWLSQWEELPPEP	5	EHLDESRYAKQTERG		
		2	SQWEELPPEPRRSGN	6	SRYAKQTERGSSTGK	9	EEGDGMKQMGELAQQ
		3	LPPEPRRSGNEHLDE	7	QTERGSSTGKEEGDG	10	MKQMGELAQQCEGGT
		4	RRSGNEHLDESRYAK	8	SSTGKEEGDGMKQMG		
	4	1	ELAQQCEGGTYADLL	5	AVVHSVRALMLAERQ		
		2	CEGGTYADLLVEAEQ	6	VRALMLAERQNPNIL	9	GEHLNKKRVLVQRPR
		3	YADLLVEAEQAVVHS	7	LAERQNPNILGEHLN	10	KKRVLVQRPRTILSV
		4	VEAEQAVVHSVRALM	8	NPNILGEHLNKKRVL		
	5	1	VQRPRTILSVESENA	5	MLVTLICSAKSLLLG		
		2	TILSVESENATMRSY	6	ICSAKSLLLGSCMSF	9	FAGMLVGRTADVKTP
		3	ESENATMRSYMLVTL	7	SLLLGSCMSFFAGML	10	VGRTADVKTPLWDTV
		4	TMRSYMLVTLICSAK	8	SCMSFFAGMLVGRTA		
	6	1	DVKTPLWDTVCLLMA	3	CLLMAFCAGIVVGGV	5	VVGGVDSGEVESGET
		2	LWDTVCLLMAFCAGI	4	FCAGIVVGGVDSGEV	6	DSGEVESGETKSESN

Interestingly, T cell response to *pp38* derived peptide pool 4 was detected in all line N chickens within the challenge, vaccine-challenge, and vaccine-boost groups (**Figures 7C, E, G** **)**. T cell response to *pp38* derived peptide pools 1 and 4 was induced in most line P2a chickens within the challenge, vaccine-challenge, and vaccine-boost groups (**Figures 7D, F, H**). The results indicate that *pp38* pool 4 may contain an immunodominant epitope restricted to B21 MHC haplotype, while *pp38* pools 1 and 4 may contain immunodominant epitopes restricted to B19 MHC haplotype.

### Induction of *pp38*-Specific T Cells Using the Cultured ELISPOT Assay Is Associated With MD-Resistance in Chickens

The results demonstrated that no *pp38*-specific-IFN-gamma-producing T cells were detected in the control groups of both the line N and line P2a chickens, indicating that this response is only induced in chickens infected with virulent or vaccine strains of MDV. In addition, the short-term T cell lines generated in presence of the relevant *pp38* peptide pools did not produce IFN-gamma when the cells were restimulated with the irrelevant peptide (H5_246–260_), and conversely, T cell lines generated in the presence of the irrelevant peptide did not respond to MDV peptide pools, indicating that the short-term T cell lines produce IFN-gamma in an antigen-specific manner.

**Figure 8:**
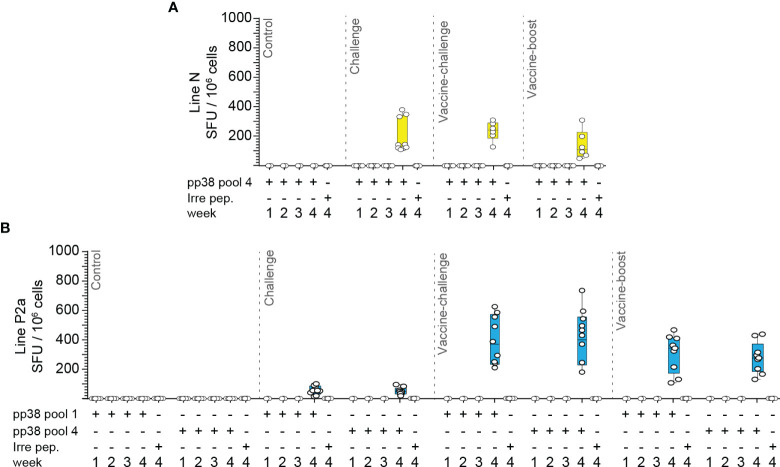
MDV infection of naïve line N chickens induces higher frequencies of IFN-gamma producing *pp38*-specific T cells compared to that in line P2a chickens. Mononuclear cells isolated from different experimental groups (Control, Infected, vaccine-challenge, and vaccine-boost) were cultured in the presence of rchIL-2 and the *pp38* peptide pools 1, 4 or an irrelevant peptide for 9–10 days. Antigen-specific IFN-gamma producing T cells were analyzed using cultured chIFN-gamma ELISPOT assay at days 9–10 post culture. The frequency of antigen specific IFN-gamma producing T cells (SFU/10^6^ cells) are presented for weeks 1–4 post the last inoculation from the **(A)** line N and **(B)** line P2a chicken lines. All ELISPOT assays were performed in triplicates and the results are representative of at least 6 chickens within each experimental group from either the line N or line P2a chickens. The results are presented as SFU per 10^6^ cells and shown as mean ± SD.

In contrast to the results obtained at 4 wpi, no *pp38*-specific IFN-gamma-producing cells were detected at 1, 2 or 3 wpi in the challenge, vaccine-challenge, and vaccine-boost groups (**Figures 8A, B** **)**. Interestingly, at 4 wpi, in the challenge group, the frequencies of *pp38*-specific T cells in line N chickens (**Figure 8A**) were significantly higher (p <0.001) than that in line P2a chickens (**Figure 8B**). In contrast, there was no significant difference in the frequencies of *pp38*-specific-IFN-gamma-producing cells in the vaccine-challenge and vaccine-boost groups between line N (**Figure 8A**) and line P2a chickens (**Figure 8B**). Taken together, the results indicate that, in line P2a chickens, vaccination induces potent T cell responses, while infection of naïve birds with virulent MDV fails to induce a potent *pp38*-specific IFN-gamma producing T cells.

## Discussion

A striking difference in virus-specific T cell responses between two genetically defined naïve chicken lines, B19 and B21 haplotypes, was observed after MDV infection using the cultured, but not the *ex vivo* standard, IFN-gamma ELISPOT assays. This may suggest that naïve B21 MHC haplotype chickens are more efficient in induction of anti-MDV central memory T cells following MDV infection. However, it is unclear whether *pp38* or *MEQ*-specific IFN-gamma producing T cells play any role in providing protection against MD. IFN-gamma produced by tumor-specific CD4 and CD8 T cells prime macrophages, which can directly eliminate tumor cells (29). The role of chIFN-gamma in providing protection against MDV-induced lymphoma has been documented (18, 19), however, further studies are required to determine the exact mechanism involved in IFN-gamma-induced protection in the MDV-infected chickens.

It is yet unknown why naïve line P2a birds cannot mount an effective T cell response to MDV detected by the cultured ELISPOT assay at 4 wpi. While this response can be induced in the vaccinated line P2a birds or naïve line N chickens after MDV infection. Treg cells can inhibit the differentiation of IFN-gamma-producing Th1 cells from naïve T cells and suppress signaling pathway and transcription factors that are involved in the induction of IFN-gamma (30, 31). Chicken TGF-beta+ Treg cells are expanded in the transformation phase of the disease in the MD-susceptible line P2a chickens, but not in the MD-resistant line N chickens, and thus an association between the induction of TGF-beta+ Treg cells and host susceptibility to MD has been suggested (32). It is possible, but not proven, that the expansion of MDV-induced chicken TGF-beta+ Treg cells may be involved in modulation of MDV-specific-IFN-gamma-producing T cells which are detected by the cultured ELISPOT assay (33). If this is the case, it is expected that TGF-beta+ Treg cells should not be expanded in the vaccinated line P2a chickens after MDV infection. These experiments are planned with the aim to identify the effects of immunoregulatory and suppressive pathways in modulation of MDV-specific T cell responses.

It had been shown that cytotoxic T cell response to lytic antigen *pp38* and latency/transformation antigen *MEQ* can be detected in MDV-infected chickens (13), however, the role of T cell response to *pp38* and *MEQ* in control of MD is currently unknown. MDV contains over 85 different viral proteins, and analysis of T cell responses to all these antigens was not feasible in this study. *pp38* and *MEQ* were selected based on their differential levels of immunogenicity in MDV-infected chickens (12–15) and practical aspects regarding the size of proteins which impact on numbers of peptides being synthesized and most importantly the number of cells required to analyze against peptide pools. *pp38* and *MEQ* are expressed in both virulent MDV and CVI988-Rispens (vaccine strains of MDV used in this study), however, CVI988-Rispens expresses *MEQ*, which has two and four amino acid differences in the DNA binding and transactivation domains, respectively (34). Additionally, CVI988-Rispens encodes a longer form of *MEQ*, which has a proline rich repeat in the transactivation domain (35), however, the impact of these differences on anti-*MEQ-*specific-IFN-gamma-producing T cell responses is yet unknown. As splenocytes isolated from line N chickens within the vaccine-boost group responded to *MEQ*-derived peptides and produced high levels of IFN-gamma, it is very unlikely that the minor differences in the *MEQ* sequences between virulent and vaccine strains of MDV can explain the lack of *MEQ-*specific T cell response in the vaccine-boost group from line P2a chickens. Another interesting finding was that *pp38* was more immunogenic than *MEQ* in all the experimental groups. A possible explanation for lower frequencies of *MEQ*-specific-IFN-gamma-producing T cells compared to the response against *pp38* might be due to differential expression levels of these viral antigens during MDV infection (36–39). Therefore, it is possible that the differential levels of *pp38* and *MEQ* expression by infected cells (e.g., antigen presenting cells) influence the differential T cell responses against these two antigens.

Here, two types of ELISPOT assays, standard and cultured, were utilized to analyze MDV-specific T cells in chickens. To our knowledge, this is the first report in which a cultured ELISPOT assay has been used to analyze antigen-specific-IFN-gamma-producing T cell response in chickens, and we believe that the methods described in this study can be used to study chicken T cell responses to other pathogens. IFN-gamma response peaked at two weeks post-inoculation using *ex vivo* ELISPOT assay, while in the cultured ELISPOT assay, it peaked at four weeks post inoculation. This could be explained by different T cell responses, effector memory and central memory cells, respectively. This agrees with the data obtained from human T cells demonstrating that the standard IFN-gamma ELISPOT assay detects effector T cells, while T cells generated from the cultured ELISPOT assay are derived from CCR7+ or CD62L+ central memory T cells (20, 21). Antigen-specific-IFN-gamma-producing T cells, detected by the cultured ELISPOT assay, are shown to be crucial feature in protection from infectious diseases in human and murine models (28, 40–43), while this relationship has not been examined in birds. Unfortunately, no specific antibody was available for identification of central memory chicken T cells (e.g., CCR7+ or CD62L+ T cells) to determine whether T cells detected by the cultured chIFN-gamma ELISPOT assay are, in fact, central memory T cells, therefore, we referred to these cells as virus-specific-IFN-gamma-producing T cells detected by the cultured ELISPOT assay. A better understanding of chicken T cells would benefit vaccine development against infectious diseases in chickens, especially in search for vaccination strategies for induction of durable immunity and correlates of protection against intracellular pathogens such as economically important viral infections. Analyzing the breadth of T cell responses, we observed that *pp38-*derived peptide 4 stimulated IFN-gamma production from splenocytes of all the experimental groups from both B19 and B21 MHC haplotype chickens, suggesting that the peptide pool 4 contains immunodominant peptides recognized by both B19 and B21 haplotype chickens. Moreover, peptide pool 1 contained an immunodominant epitope recognized by B19 MHC haplotype chickens. We have performed extensive experiments to identify the immunodominant epitopes within pools 1 and 4 and have further characterized functional abilities of T cells recognizing these immunodominant epitopes in both chicken lines. Our unpublished results (manuscript in preparation) demonstrate that T cells from B19 and B21 haplotypes recognize different peptide epitopes with no overlapping sequence.

In conclusion, our results demonstrate that MDV infection induces a differential MDV-specific-IFN-gamma-producing T cells in chickens with B19 and B21 MHC haplotypes, suggesting that there is an association between induction under certain conditions of at least one kind of virus-specific T cell and resistance to the disease.

## Data Availability Statement

The original contributions presented in the study are included in the article/**Supplementary Material**. Further inquiries can be directed to shahriar.behboudi@pirbright.ac.uk.

## Ethics Statement

The animal study was reviewed and approved by Animal Welfare and Ethical Review Body at The Pirbright Institute.

## Author Contributions

Conceptualization: SB. Data curation: NB and SB. Formal analysis: NB and SB; Funding acquisition: SB. Investigation: NB and SB. Methodology: NB and SB. Supervision: SB Visualization: NB, SB. Writing—Original draft: NB and SB. Writing—Review & editing: SB. All authors contributed to the article and approved the submitted version.

## Funding

This work was supported by U.K. Research and Innovation Biotechnology and Biological Sciences Research Council Grants BBS/E/I/00001825, BBS/E/I/00007030, BBS/E/I/00007031, BB/S01506X/1, BBS/E/I/00002529, BBS/E/I/00007039, BBS/E/I/00007032, BB/N002598/1 and BB/V019031/1.

## Conflict of Interest

The authors declare that the research was conducted in the absence of any commercial or financial relationships that could be construed as a potential conflict of interest.

## Publisher’s Note

All claims expressed in this article are solely those of the authors and do not necessarily represent those of their affiliated organizations, or those of the publisher, the editors and the reviewers. Any product that may be evaluated in this article, or claim that may be made by its manufacturer, is not guaranteed or endorsed by the publisher.

## Supplementary Material

Supplementary Figure 1 Representative of ELISPOT wells. ELISPOT images from splenocytes stimulated with *pp38* derived peptides, *MEQ* derived peptides, Phorbol Myristate Acetate (PMA; 50 ng/ml) plus Ionomycin (ION; 1 μg/ml) (positive control) and negative control (-neg.) are shown.
