# Supplementary figures and images for: Differential Virus-Specific IFN-Gamma Producing T Cell Responses to Marek’s Disease Virus in Chickens With B19 and B21 MHC Haplotypes
Source: Front Immunol. 2022 Jan 13;12:784359. doi: 10.3389/fimmu.2021.784359 (PMC8792850; doi:10.3389/fimmu.2021.784359)

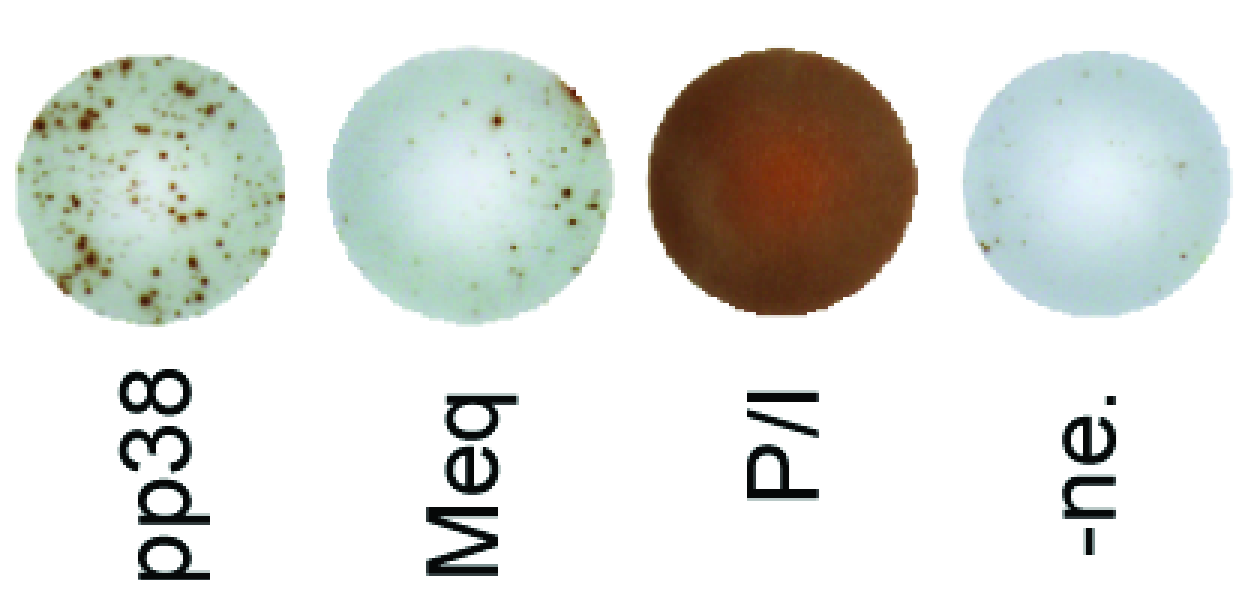

Supplement: Supplementary Figure 1 — Representative of ELISPOT wells. ELISPOT images from splenocytes stimulated with pp38 derived peptides, MEQ derived peptides, Phorbol Myristate Acetate (PMA; 50 ng/ml) plus Ionomycin (ION; 1 μg/ml) (positive control) and negative control (-neg.) are shown. [file Image_1.tif]
